# Modeling Brownian Microparticle Trajectories in Lab-on-a-Chip Devices with Time Varying Dielectrophoretic or Optical Forces

**DOI:** 10.3390/mi12101265

**Published:** 2021-10-18

**Authors:** Mohammad Asif Zaman, Mo Wu, Punnag Padhy, Michael A. Jensen, Lambertus Hesselink, Ronald W. Davis

**Affiliations:** 1 Department of Electrical Engineering, Stanford University, Stanford, CA 94305, USA; mowu@stanford.edu (M.W.); punnag@stanford.edu (P.P.); hesselink@ee.stanford.edu (L.H.); 2 Stanford Genome Technology Center, Department of Biochemistry, Stanford University, Palo Alto, CA 94304, USA; m.a.jensen@stanford.edu (M.A.J.); dnamarkr@stanford.edu (R.W.D.); 3 Department of Genetics, Stanford University, Palo Alto, CA 94304, USA

**Keywords:** Brownian dynamics, Lab-on-a-chip, Langevin equation, dielectrophoresis, optical trap

## Abstract

Lab-on-a-chip (LOC) devices capable of manipulating micro/nano-sized samples have spurred advances in biotechnology and chemistry. Designing and analyzing new and more advanced LOCs require accurate modeling and simulation of sample/particle dynamics inside such devices. In this work, we present a generalized computational physics model to simulate particle/sample trajectories under the influence of dielectrophoretic or optical forces inside LOC devices. The model takes into account time varying applied forces, Brownian motion, fluid flow, collision mechanics, and hindered diffusion caused by hydrodynamic interactions. We develop a numerical solver incorporating the aforementioned physics and use it to simulate two example cases: first, an optical trapping experiment, and second, a dielectrophoretic cell sorter device. In both cases, the numerical results are found to be consistent with experimental observations, thus proving the generality of the model. The numerical solver can simulate time evolution of the positions and velocities of an arbitrarily large number of particles simultaneously. This allows us to characterize and optimize a wide range of LOCs. The developed numerical solver is made freely available through a GitHub repository so that researchers can use it to develop and simulate new designs.

## 1. Introduction

Recent decades have seen rapid advances in the field of biotechnology. Lab-on-a-chip (LOC) devices capable of manipulating micro- and nano-sized bio-samples have played a significant role in fueling this progress. The research on designing novel and complex LOC devices is ongoing. A computational-physics framework capable of simulating the trajectory of a micro- or nano-sample can be a very useful tool for designing and analyzing LOCs [1,2].

Although the main application of LOCs is in the field of biology, the design process of the devices requires modeling several physical phenomena. Trapping and manipulating small-sized samples are often a key feature of LOCs. On-chip manipulation of micro-sized samples (or microparticles) is usually accomplished by using viscous drag forces applied through fluid flow along with dielectrophoresis (DEP) [3,4,5] or optical [6,7,8]/ optoelectronic [9,10,11] techniques (DEP is more widely used). LOCs can have integrated micro-electrodes and microfluidic channels to carry out these functions [12]. Designing a device to achieve a specific functionality requires accurately predicting how a micro-object behaves under the influence of relevant forces. For example, microfluidic particle/cell sorter devices often utilize material-selective DEP forces to create separate trajectories for different types of particles (e.g., dead vs live cells) [13,14,15,16,17,18]. The design process hinges on engineering these trajectories. Similar arguments can be made for LOC devices used in other applications as well. A mathematical simulation model capable of predicting particle trajectories can help designers identify potential issues and optimize the device before proceeding to the fabrication step; consequently, saving time and cost.

Along with the effects of external forces, colloidal particles (we use the term *particle* throughout the paper to refer to any colloidal micro- or nano-specimen of roughly spherical shape) in LOCs exhibit Brownian motion which can affect their trajectories [19]. Brownian motion arises from random collisions between the particle and the molecules of the suspension fluid [20,21]. Although the phenomenon is usually not prominent for large particle sizes (tens of microns) [18], it can be notable when sizes are in sub-micron range. Therefore, it should be included in a generalized model. The Langevin equation, which takes into account the effects of external forces and Brownian motion, can be used for this purpose [20,22]. The viscous drag force experienced by the moving particle in the suspension fluid (which itself can be in motion) is integrated within the equation. This makes it ideal for modeling colloidal particle trajectories in a static or moving fluid.

Unlike colloids in a boundless isotropic fluid, particles in LOC devices are bounded within a finite-sized liquid chamber. As such, additional mechanics need to be taken into account for modeling particle behavior. Firstly, a particle close to the walls of the fluid chamber or a microfluidic channel would experience *hindered* diffusion instead of free diffusion [23,24,25]. This is due to the hydrodynamic interaction between the particle and the no-slip boundary layer [2] of the fluid near a solid surface. Another factor to consider is particle collision with the solid surfaces of the chip which alters its motion. In multi-particle systems, particle–particle collisions are also possible. The effect of collisions cannot be directly included within the Langevin equation and therefore, must be modeled separately. In this work, we present a numerical model that takes into account all the aforementioned physics to calculate particle trajectories inside LOCs. The numerical solver uses a finite-difference scheme to solve the Langevin partial-differential equation and obtain the time evolution of particle positions. We model hindered diffusion by using a particle-position dependent diffusion tensor in the Langevin equation. A collision detection algorithm is used at each time-step. If detected, an elastic collision model is used to modify the results of the Langevin mechanics. Our generalized model is capable of simulating heterogeneous multi-particle systems, where each particle can differ in size, mass, and material properties. In addition, a large number of particles can be simulated simultaneously. The numerical solver computes the particle positions in parallel and, therefore, does not require long computation time even for large number of particles. Thus, it is ideal for simulating and analyzing various different LOC environments.

The numerical solver is implemented in python and is made available publicly through a GitHub repository [26]. We chose python as it is open source and has many freely available scientific-computing libraries. The software and the mathematical libraries required to run the solver are freely available. Along with the numerical engine, we utilized the graphical libraries to create built-in visualization schemes for analyzing relevant data. This includes the ability to save the animation of the particle motion in the form of a video file and logging the position and velocity values of each particle.

To demonstrate the usefulness of the platform, we present simulation results of two LOC systems. The first example shows optical trapping of multiple microparticles. A dynamic nature is incorporated by activating and deactivating the trap at specific time instances. The corresponding simulation results show the trapping mechanics, as well as the dynamics of the particles when the trap is released. The second example covers a more complex LOC that uses DEP to sort and separate cells. Such LOCs have many applications [12,14,15]. A microfluidic device with flowing liquid is considered for this case. Material selective DEP force is used to direct viable (live) and non-viable (dead) yeast cells along different paths. These two distinctly different examples show the versatility of our solver for simulating various LOC configurations. Moreover, the solver is developed in modular form. Thus, switching from one device geometry to another requires modifying only a few modules and plugging in external force terms.

The rest of the paper is organized as follows: Section 2 introduces the Langevin equation and its discretization to the finite difference form. The particle position dependent diffusion tensor and collision mechanics are discussed in Section 3 and Section 4, respectively. Section 5 describes how all the physics were integrated in the numerical solver. The simulation results for the two different LOCs are discussed in Section 6. Finally, concluding remarks are made in Section 7.

## 2. Langevin Equation

The motion of a colloidal micro- or nano-particle under the influence of an external force-field can be modeled using the Langevin equation. For a colloidal particle in a low Reynolds number environment with shear-flow, the Langevin equation has the form [20,27,28,29]:

(1) $$m\frac{\partial v(r,t)}{\partial t}=\frac{k_{B}T}{\overset{↔}{D}\left(r\right)}\left[v_{f}\left(r,t\right)-v\left(r,t\right)+\sqrt{2}{\overset{↔}{D}}_{\frac{1}{2}}\left(r\right)W\left(t\right)\right]+F_{\mathrm{ext}}\left(r,t\right)\;.$$

Here, *t* is the time variable, *m*, r and v(r,t) are the mass, position, and velocity of the particle, respectively, $v_{f}\left(r,t\right)$ is the velocity of the fluid, $k_{B}$ is the Boltzmann constant, *T* is the temperature, $F_{\mathrm{ext}}$ is the net external applied force acting on the particle, $\overset{↔}{D}$ is the diffusion tensor, and W(t) is a vector white noise term. The tensor ${\overset{↔}{D}}_{\frac{1}{2}}$ is defined as the element-wise square root of $\overset{↔}{D}$. Each Cartesian component of W(t) is a random process with zero mean and unit variance. The $m\dot{v}\left(r,t\right)$ term represents the inertia of the particle. This term can often be dropped for small particles where the mass is negligible [20]. The friction/drag force term, $\left[v_{f}\left(r,t\right)-v\left(r,t\right)\right]k_{B}T/\overset{↔}{D}$, depends on the viscous properties of the fluid [17]. The white noise term $\sqrt{2}{\overset{↔}{D}}_{\frac{1}{2}}\left(r\right)W\left(t\right)$ is the fluctuating force resulting from random collisions between the particle and fluid molecules. The $F_{\mathrm{ext}}$ term encapsulates all external forces acting on the particle (e.g., optical trapping force, dielectrophoretic force, gravitational force, etc.). Whereas most research articles use the derivatives of the position vector, we express Equation (1) in terms of the velocity vector and its derivative. We do so for easier subsequent integration of collision physics into the model which depends on the velocity vectors. Thus, directly solving the velocity vectors simplifies the analysis.

Equation (1) is a stochastic differential equation. The presence of the discontinuous white noise term makes solving a stochastic differential equation more complex than solving an ordinary differential equation [30]. Our approach to numerically solve Equation (1) involves using the Euler–Maruyama method to convert it into a finite difference equation [30,31]. We use the following discretization scheme:

(2) $$m\frac{v_{i+1}-v_{i}}{\Delta t}=\frac{k_{B}T}{\overset{↔}{D}\left(r_{i}\right)}\left[v_{f,i}-v_{i+1}+\sqrt{\frac{2}{\Delta t}}{\overset{↔}{D}}_{\frac{1}{2}}\left(r_{i}\right)w_{i}\right]+F_{\mathrm{ext},i}\left(r_{i}\right)\;.$$

Here, $i=0,1,\cdots N_{t}$ represents the time index and Δt is the time step. The set of discrete time where the numerical solution is calculated is given by $t_{i}=i\Delta t$. The term $w{}_{i}$ is a random vector whose Cartesian components are Gaussian random numbers with zero mean and unit variance. Solving for $v_{i+1}$ from Equation (2) gives:

(3) $$v_{i+1}=\frac{\overset{↔}{\Lambda }}{1+\overset{↔}{\Lambda }}v_{i}+\frac{1}{1+\overset{↔}{\Lambda }}\left[v_{f,i}+\sqrt{\frac{2}{\Delta t}}{\overset{↔}{D}}_{\frac{1}{2}}\left(r_{i}\right)w_{i}+\frac{\overset{↔}{D}\left(r_{i}\right)}{k_{B}T}F_{\mathrm{ext},i}\left(r_{i}\right)\right]\;.$$

Here, $\overset{↔}{\Lambda }≜\frac{m\overset{↔}{D}}{k_{B}T\Delta t}$ is a unit-less tensor quantity defined to make the equation compact. It should be noted that we use $v_{i+1}$ instead of $v_{i}$ in the right hand side of Equation (2). This ensures the stability of the numerical scheme for arbitrary Δt values. This fact can be verified by setting the fluid velocity, the white noise term, and the external force to be zero and observing the simplified velocity update equation: $v_{i+1}=\frac{\overset{↔}{\Lambda }}{1+\overset{↔}{\Lambda }}v_{i}$. As the multiplicative factor is less than unity (every component of $\overset{↔}{\Lambda }$ is positive), the velocity will remain bounded over successive iterations and thus, stay stable.

After calculating the velocity vector at a given time step *i*, the position vector can be easily calculated using the Euler–Cromer method [32]:

(4) $$r_{i+1}=r_{i}+v_{i+1}\Delta t\;.$$

The position and velocity data of each particle is calculated using the same equations. The process is repeated for the next discrete time instance until the simulation end time is reached.

## 3. Diffusion Tensor and Hydrodynamic Interactions

The Langevin equation contains the diffusion tensor which is a representation of the fluid–particle interaction. The diffusion tensor in an isotropic homogeneous medium, ${\overset{↔}{D}}_{0}$, depends on the material properties of the suspension medium, particle size, and the temperature. It is given by [23,24]:

(5) $${\overset{↔}{D}}_{0}=\frac{k_{B}T}{6\pi \eta r_{o}}\overset{↔}{I}\;,$$

where $\overset{↔}{I}$ is a 3×3 unit tensor, $r_{o}$ is the radius of the particle, and η is the dynamic viscosity of the medium. Equation (5) is equivalent to using a scalar diffusion coefficient. For LOC devices, however, the fluid chamber or microfluidic channels are not large enough compared to the particle size for assuming isotropic conditions. In addition, particles are often located near the bottom surface during manipulation in such devices. So, the particle dynamics are different from the isotropic case. The hydrodynamic interactions between the particle and the static fluid layers near a surface hinder the diffusion process. Thus, the diffusion tensor becomes dependent on the relative position of the particle with respect to the solid surfaces of the device. The generalized diffusion tensor for such cases is given by [23,24]:

(6) $$\overset{↔}{D}\left(r\right)=\frac{k_{B}T}{6\pi \eta r_{o}}\overset{↔}{H}\left(r\right)\;.$$

$\overset{↔}{H}\left(r\right)$ is a 3×3 unitless tensor that depends on the distance of the particle from the solid surfaces. For a geometry aligned with the coordinate system, only the diagonal elements of the tensor will be non-zero, i.e., $\overset{↔}{H}\left(r\right)=\left[H_{jk}\left(r\right)\right]$, $H_{jk}\left(r\right)=0$∀j≠k.

The majority of the hydrodynamic interactions in LOCs arise from the bottom/top surface along which particle manipulation takes place. Thus, we consider a case with a single solid surface located at the z=0 plane. The components of $\overset{↔}{H}\left(r\right)$ can be expressed as $H_{11}\left(r\right)=H_{22}\left(r\right)=H_{\|}\left(r\right)$ and $H_{33}\left(r\right)=H_{⊥}\left(r\right)$ where [23,24,25]:

(7) $$\begin{array}{c} H_{\|}\left(r\right)=1-\frac{9r_{o}}{16z}+\frac{r_{o}^{3}}{8z^{3}}-\frac{45r_{o}^{4}}{256z^{4}}-\frac{r_{o}^{5}}{16z^{5}}, \end{array}$$

(8) $$\begin{array}{c} H_{⊥}\left(r\right)=\frac{6z^{2}+2r_{o}z}{6z^{2}+9r_{o}z+2r_{o}^{2}}\;. \end{array}$$

**Figure 1:**
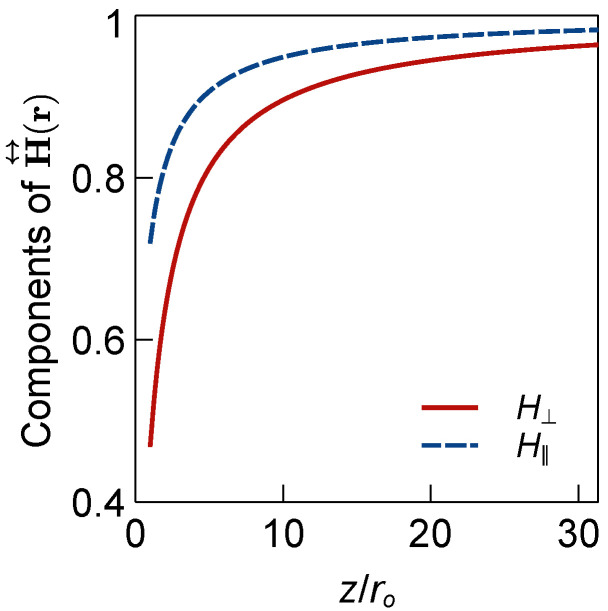
Components of $\overset{↔}{H}\left(r\right)$ as functions of $z/r_{o}$ considering a solid surface located at the z=0 plane.

Here, *z* is the *z*-position of the particle. In Figure 1, $H_{\|}\left(r\right)$ and $H_{⊥}\left(r\right)$ are plotted as functions of the normalized variable $z/r_{o}$. It can be observed that for particle position far from the z=0 surface (i.e., high $z/r_{o}$ values), the value of both terms approach unity and the diffusion tensor approaches the free space diffusion tensor. The closer the particle is to the surface, the larger the variation is from the free space case. Values less than unity suggests that the diffusion process is hindered near solid surfaces.

## 4. Collision Mechanics

In a multi-particle system, particle-particle collisions need to be taken into account when simulating particle trajectories. We consider a system with $N_{p}$ particles where particle *p* and particle *q* collide at time instance *i*. Their mass, position, and initial velocity are $m_{p}$, $m_{q}$, $r_{p,i}$, $r_{q,i}$, and $v_{p,i}$, $v_{q,i}$, respectively. The velocity vectors after the collision, $v_{p,i}^{\mathrm{col},\mathrm{pp}}$ and $v_{q,i}^{\mathrm{col},\mathrm{pp}}$, can be calculated using the theory of elastic collisions where the kinetic energy and momentum of the particles are conserved.

The collision mechanics in three-dimensional systems can be treated by considering the velocities along the normal and tangential directions separately. We note that the velocity along the normal direction of the colliding surfaces are altered after collision, whereas the tangential velocity components are unaffected [33]. First, we define the normal vector:

(9) $$\hat{n}=\frac{r_{p,i}-r_{q,i}}{|r_{p,i}-r_{q,i}|}\;.$$

The normal and tangential component of the particle *p* velocity is then given by $\left(v_{p,i}\;\cdot \;\hat{n}\right)\hat{n}$ and $v_{p,i}-\left(v_{p,i}\;\cdot \;\hat{n}\right)\hat{n}$, respectively. One-dimensional elastic collision formula can be applied for the normal directions. The velocity after the collision for particle *p* is given by:

(10) $$v_{p,i}^{\mathrm{col},\mathrm{pp}}=\frac{\left(v_{p,i}\;\cdot \;\hat{n}\right)\hat{n}\left(m_{p}-m_{q}\right)+2m_{q}\left(v_{q,i}\;\cdot \;\hat{n}\right)\hat{n}}{m_{p}+m_{q}}+v_{p,i}-\left(v_{p,i}\;\cdot \;\hat{n}\right)\hat{n}\;.$$

It can be noted that the first term of Equation (10) is identical to the 1D elastic collision formula along the normal axis. Simplifying Equation (10) gives:

(11) $$v_{p,i}^{\mathrm{col},\mathrm{pp}}=v_{p,i}-\frac{2m_{p}}{m_{p}+m_{q}}\left[\left(v_{p,i}-v_{q,i}\right)\;\cdot \;\hat{n}\right]\hat{n}$$

Swapping the subscripts *p* and *q* in Equation (11) gives the modified velocity equation for particle *q*.

In addition to particle–particle collisions, collisions with a wall/solid-surface may also occur in LOC systems (e.g., collision with bottom substrate, sidewalls of microfluidic channels, etc.). Assuming that the walls are much more massive than the particles, the velocity of a particle after collision can be calculated using a reflection operation. If particle *p* collides with a flat wall with surface normal ${\hat{n}}_{\mathrm{col},\mathrm{pw}}$, then the velocity is modified as:

(12) $$v_{p,i}^{\mathrm{col},\mathrm{pw}}=v_{p,i}-2\left(v_{p,i}\;\cdot \;{\hat{n}}_{\mathrm{col},\mathrm{pw}}\right){\hat{n}}_{\mathrm{col},\mathrm{pw}}$$

Using Equations (11) and (12), most common collision mechanics occurring in LOC environments can be modeled.

## 5. Physics Integration

We incorporate all the aforementioned physics into the particle dynamics solver. The solver starts with randomly populated position and velocity vectors for each particle. A vector white noise term is generated from a normal distribution. The particle position depended diffusion tensor is calculated from Equation (6). Then Equation (3) and (4) is used to find predicted particle velocity and position for the next time step (i.e., $v_{i+1}$ and $r_{i+1}$, respectively). As the Langevin equation does not take into account the presence of any obstacles in the particle path, the calculated position vector may represent an unphysical condition (i.e., two particles overlapping, particles going through walls, etc.). To correct for this, the Euclidean distances between each particle pair and each particle-wall pair are calculated to check for collisions. If a collision is detected, then position and velocity given by the Langevin equations needs to be adjusted. Using Equation (11) or Equation (12), the velocity vector is adjusted as:

(13) $$v_{p,i+1}^{\mathrm{adj}}=\left\{\begin{array}{cc} v_{p,i+1}^{\mathrm{col},\mathrm{pp}}, & \mathrm{if}\;\mathrm{collision}\;\mathrm{with}\;\mathrm{particle}.\;\\ v_{p,i+1}^{\mathrm{col},\mathrm{pw}}, & \mathrm{if}\;\mathrm{collision}\;\mathrm{with}\;\mathrm{wall}. \end{array}\right.$$

The corresponding adjusted position vector is:

(14) $$r_{p,i+1}^{\mathrm{adj}}=r_{p,i}+v_{p,i+1}^{\mathrm{adj}}\Delta t\;.$$

**Figure 2:**
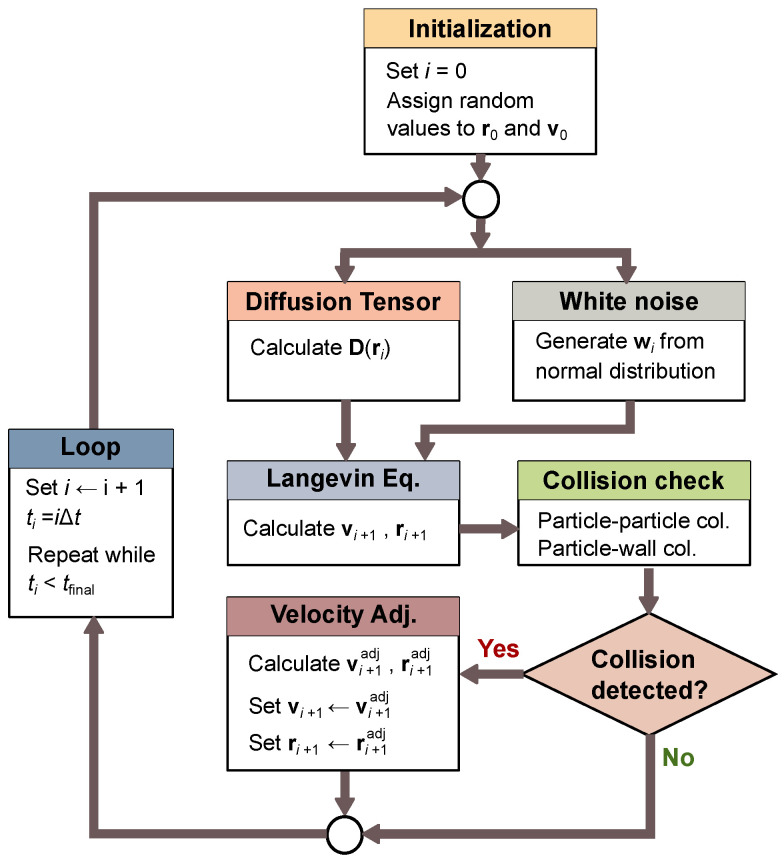
Flowchart of particle dynamics simulation steps.

The velocity and position vectors are replaced by the adjusted quantities (i.e., $v_{p,i+1}\leftarrow v_{p,i+1}^{\mathrm{adj}}$ and $r_{p,i+1}\leftarrow r_{p,i+1}^{\mathrm{adj}}$) before proceeding to the next time step. The Python code implements the entire process in parallel for all the particles (i.e., $p=0,1,\cdots N_{p}-1$) inside a single time loop. The implemented wall collision physics is general and works for any arbitrary polygon geometry. The flowchart shown in Figure 2 shows all the calculation steps.

## 6. Results

Using the developed algorithm, we present simulation results of two example LOC setups. The first example is a typical optical trapping setup with a Gaussian beam. The second example is a microfluidic LOC device that uses dielectrophoresis to sort or separate live and dead yeast cells.

### 6.1. Optical Trap

We consider an optical trapping experiment with a Gaussian beam. We implement a time dependent external force, modeling an optical beam that is turned ON and OFF at specific time instances. For simplicity, we assume a conservative gradient-force profile resulting from a Gaussian potential well, u(r), centered around the origin. The potential is defined as:

(15) $$u\left(r\right)=\frac{4}{3}\pi r_{o}^{3}A_{d}e^{-{\left(\frac{r}{w}\right)}^{2}}\;.$$

Here, $A_{d}=120k_{B}T$ $\mu m^{-3}$ is the depth of the volume density of the potential well (i.e., depth of the potential well normalized with respect to particle volume), $r_{o}$ is the radius of a particle, w=10 μm is representative of the width of the well, and *r* is the radial distance from the trap center (assumed to be at the origin) to the particle position. The corresponding force profile, $F_{\mathrm{opt}}\left(r\right)$, is given by [7,34]:

(16) $$F_{\mathrm{opt}}\left(r\right)=-\nabla u\left(r\right)\;.$$

**Figure 3:**
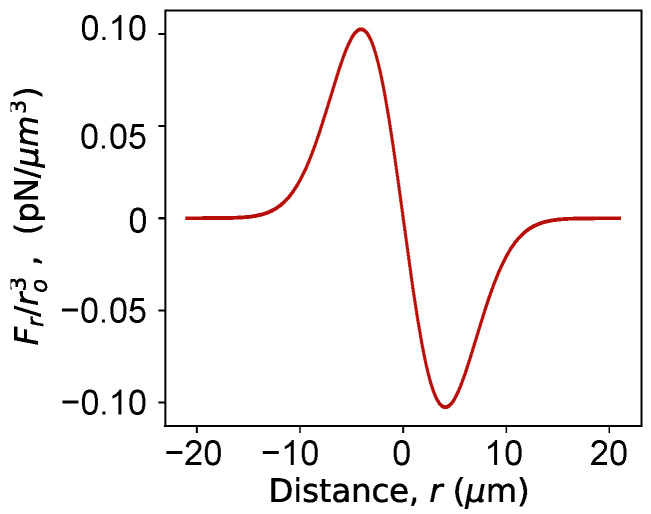
Force profile for the optical trap with Gaussian potential well.

The force profile is shown in Figure 3. Due to the symmetry of the potential well, we only plot the radial component of the force, $F_{r}$. The *x*, *y* and *z* components of the force can easily be calculated from Equation (16) as well. Both the potential profile and the force profile are assumed to scale linearly with particle volume. This is a commonly used approximation for optical traps [35]. The conservative force-profile approximation is also commonly accepted for most cases. However, for some cases, especially involving near-field trapping, it is possible to have non-conservative force components [7]. Instead of Equation (16), more generalized approaches are necessary to model the force for those cases. For the current case, we define the $F_{\mathrm{ext}}$ term in the particle dynamics model as:

(17) $$F_{\mathrm{ext}}\left(r,t\right)=\left\{\begin{array}{cc} F_{\mathrm{opt}}\left(r\right), & \mathrm{if}\;1\;s<t<8\;s\;.\;\\ 0, & \mathrm{otherwise}. \end{array}\right.$$

This represents a case when the optical beam is ON during the time interval 1 s<t<8 s. The interval boundaries are set arbitrarily. It should be noted that other possible external force terms (e.g., gravitational sedimentation, thermophoresis, etc.) can also be added here.

**Figure 4:**
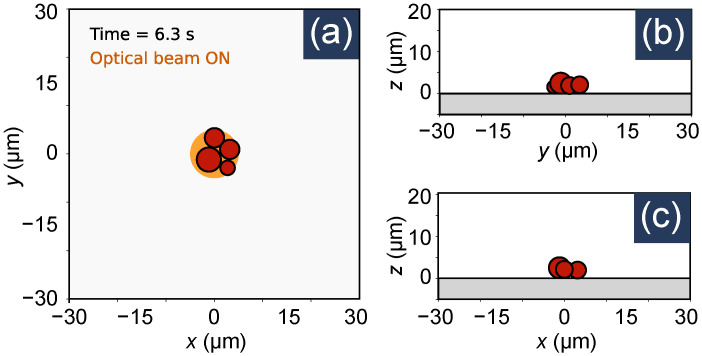
Position of particles in an optical trap at the time instance t=6.3 s. (**a**) xy plane, (**b**) yz plane, and (**c**) xz plane view. The yellow circle in (**a**) indicates the optical spot. Multimedia file 1 is an animated video that shows the particle positions at different time instances.

**Figure 5:**
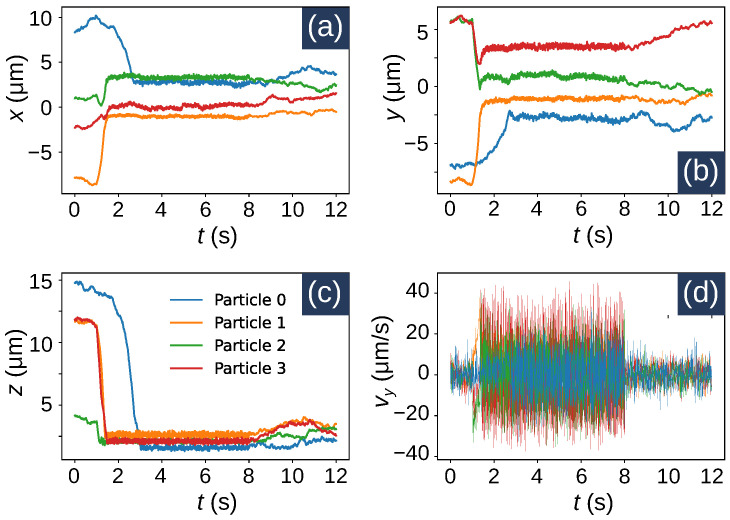
Position and velocities of particles in an optical trap as a function of time. (**a**) *x* position, (**b**) *y* position, (**c**) *z* position, and (**d**) *y* velocity component as functions of time. All the plots share the same legend.

We consider $N_{p}=4$ polystyrene beads (density, $\rho =1055\;\mathrm{kg}/m^{3}$) suspended in water (dynamic viscosity, $\eta =8.9\times 10^{-4}\;\mathrm{Pas}$, and temperature, T=300 K) with radii $r_{o,0}=1.5\;\mu m$, $r_{o,1}=2.5\;\mu m$, and $r_{o,2}=r_{o,3}=2\;\mu m$. The corresponding particle masses can be calculated from the volume and density values. A solid bottom surface is assumed to be located at the z=0 plane. With these conditions, the simulation was run from t=0 to t=12 s with a step size of Δt=0.01 s. The simulations results are shown in Figure 4 and Figure 5. From Figure 4, we can see the particle positions at an arbitrary time instance (t=6.3 s), when they are trapped on the optical spot. The associated multimedia file 1 is an animated video that shows the same results for all time instances. Figure 5 show the positions and velocities of the particles as a function of time. The position plots show that the particles start moving towards the origin at around t=1 s, when the Gaussian optical spot (centered around the origin) is turned ON. As the particles move towards the optical spot, they start colliding with each other. This is represented by the spiky nature of the plots during the time interval from t≈2 s to t≈8 s. After t>8 s, the optical spot is turned OFF and the particles are no longer trapped. During this time the Brownian motion dominates as the particles move randomly. It should be noted that a particle does not always become trapped when the optical spot is active. Trapping occurs only when a particle is within the capturing range of the trap [36,37]. In other instances of the simulation, where the random initial position of a particle is significantly away from the optical spot, the particle does not become trapped.

### 6.2. Dielectrophoretic Cell Sorting or Separation

**Figure 6:**
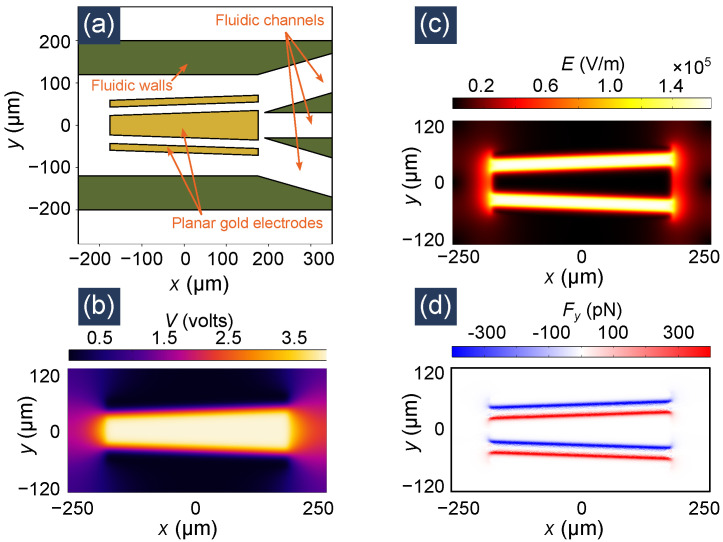
The geometry and electromagnetic simulation of the dielectrophoretic cell sorter device. (**a**) Top view of the device, (**b**) Electric potential distribution, (**c**) Electric field distribution, and (**d**) Force experienced by a viable yeast cell. All figures depict the xy plane view of the three-dimensional device. For simulation, the large middle electrode is excited with 4 V, 5 MHz AC voltage, whereas the other two electrodes are grounded.

For our second example, we simulate a microfluidic device that uses DEP forces for cell sorting or separation which is a common application of LOCs [15,16,17]. Specifically, we focus on the separation of viable (live) and non-viable (dead) yeast cells using the device proposed by Doh et al. in their paper [15]. The device uses tapered electrodes embedded within a microfluidic chamber. The structure is shown in Figure 6a. The fluid walls are 50 μm high. The electrodes having a height of 0.12 μm can be considered planar. The electrodes have a length of 350 μm (along the *x* axis) and the gap between them is 16 μm. The top and bottom electrodes have a width (along *y* axis) of 20 μm, whereas the width of the tapered middle electrode spans 46–70 μm. The top and bottom electrodes are electrically shorted externally. An AC voltage of 4 V at 5 MHz is applied between the center electrode and the other electrodes. A strong field is created between the electrodes generating dielectrophoretic forces. As a solution of cells flows from left to right, the electrodes exert different forces on live and dead cells, modifying their trajectories. Thus, specific cells can be diverted to specific microfluidic channels. We choose this device as an example as it incorporates some of essential particle manipulation mechanics (e.g., fluid flow, dielectrophoretic force, etc.) used in many LOC devices.

To use our numerical solver to simulate this device, first we have to model the forces acting on the cells. The time averaged DEP force, $F_{\mathrm{DEP}}$, on the cells can be approximated as [3,15,17]:

(18) $$F_{\mathrm{DEP}}=2\pi \epsilon_{0}\epsilon_{m}r_{o}^{3}ℜ[{\tilde{C}}_{M}]\nabla {\left|E\right|}^{2}\;.$$

Here, $\epsilon_{0}$ is the permittivity of free space, $\epsilon_{m}=80$ is the relative permittivity of the medium, $r_{o}=3\;\mu m$ [38] is the cell radius, ${\tilde{C}}_{M}$ is the Clausius–Mossotti factor, ℜ[·] is the operator that outputs the real part of its argument, and E is the electric field. E depends on the geometry of the electrodes and the device. It can be noted that the sign of $ℜ\left[{\tilde{C}}_{M}\right]$ determines whether $F_{\mathrm{DEP}}$ is attractive (positive DEP) or repulsive (negative DEP). Since ${\tilde{C}}_{M}$ depends on the material properties of the cells, they differ from live cells to dead cells [39]. We use the multi-shell model to calculate ${\tilde{C}}_{M}$ for viable and non-viable yeast cells [3]. The procedure is discussed in detail in the Supplementary Information Document. We take the geometrical and material parameters of the cells and the device dimensions from the literature [15,40,41]. Plugging in these values, we find that at f=5MHz, the viable and non-viable cells have relative permittivities of $\epsilon_{\mathrm{viable}}=199.94$, and $\epsilon_{\mathrm{nonviable}}=18.82$, respectively, and have conductivities of $\sigma_{\mathrm{viable}}=0.36\;\mathrm{S/m}$ and $\sigma_{\mathrm{nonviable}}=0.013\;\mathrm{S/m}$, respectively. These values correspond to $ℜ\left[{\tilde{C}}_{M,\;\mathrm{viable}}\right]=0.945$ and $ℜ\left[{\tilde{C}}_{M,\;\mathrm{nonviable}}\right]=-0.25$. Thus, the viable cells experience positive DEP, whereas the non-viable cells experience negative DEP.

The electric field term in Equation (18) can be calculated using any of the widely available numerical electromagnetic field solvers for any arbitrary device geometry. We use Comsol Multiphysics^®^ for this purpose. The calculated electric potential and field distributions are shown in Figure 6b,c. The corresponding force values on a viable yeast cell (calculated using Equation (18)) are shown in Figure 6d. It can be seen that the force pulls the viable cells near the gap regions between the electrodes where the field intensity is maximum. For a non-viable cell, the sign of the force would be reversed and scaled by a factor of $|ℜ\left[{\tilde{C}}_{M,\;\mathrm{nonviable}}\right]|/|ℜ\left[{\tilde{C}}_{M,\;\mathrm{viable}}\right]|\approx 0.26$.

Unlike the optical tweezers example, the cell sorter device works with a flowing fluid instead of a stationary pool of liquid. A left to right fluid flow with velocity of 150 μm/s is considered. Due to the presence of the microfluidic channels, the fluid velocity does not maintain the purely left-to-right direction throughout the device. Considering the fact that the average fluid flow direction is tangential to the channel surface, we use the following simplified piece-wise model:

(19) $$v_{f}\left(r,t\right)=\left\{\begin{array}{cc} 150\hat{x}+30\hat{y}\;, & \mathrm{if}\;x>170\mu m\;,y>30\mu m\;.\;\\ 150\hat{x}-30\hat{y}\;, & \mathrm{if}\;x>170\mu m\;,y<-30\mu m\;.\;\\ 150\hat{x}\;, & \mathrm{otherwise}. \end{array}\right.$$

Here, $v_{f}\left(r,t\right)$ has units of μm/s. The *y* component of the fluid velocity for a given *x* component is approximated from the slope of the channels so that the average flow is tangential to the fluid wall. A 3D moving average filter was applied to make the velocity profile smoother. It should be stated that this flow profile is an approximation. We also ignore any fluid flow that might be induced by the non-uniform electric field [42]. It is possible to plug in more accurate data from an external solver in our code. However, the piece-wise model is sufficiently accurate for the current demonstration problem. In the future, we plan to implement a numerical fluid flow solver and integrate it with our particle dynamics solver.

**Figure 7:**
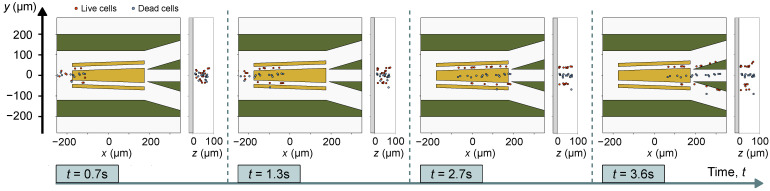
Position of live and dead yeast cells at four time instance. All figures share the same *y* axis. At each time instance, the particle position is plotted in xy and zy planes. The times instances are separated by dashed lines. The red circles represent live cells and the blue circles represent dead cells. The green regions are microfluidic walls, whereas the gold region are the planar electrodes. The gray region represents the substrate of the device. Multimedia file 2 contains an animation showing data of all simulated time instances.

From Equations (18) and (19), we plug in the $F_{\mathrm{ext}}=F_{\mathrm{DEP}}$ and the fluid flow velocity in Equations (3) and (4) to solve the particle dynamics in the time range t=0 to t=6 s with a time step of Δt=5ms. Like the optical trapping example, the particle–particle collision and particle–wall collision models are included in the simulation. We simultaneously simulate the trajectory of 17 viable yeast cells and 17 non-viable yeast cells with random initial positions located in the domain x∈[−480,−200]μm, y∈[−280,280]μm and z∈[6,80]μm. The simulation results are shown in Figure 7. Four time instances are depicted here. The entire time evolution of the cell positions can be seen in the attached multimedia file 2. Although both live and dead cells experience the same fluid flow, they experience different DEP forces as they have different $ℜ\left[{\tilde{C}}_{M}\right]$ values. We note that as the cells are carried left-to-right by the fluid flow, the live cells (depicted in red) become deflected top/bottom (near the electrode gaps) whereas the dead cells (depicted in blue) are pushed towards the center electrode. This is consistent with the force profile shown in Figure 6d. As the cells travel to the right of the device near the output fluid channels, the cells are grouped as can be seen in t=1.3 s and t=2.7 s frames of Figure 7. The top and bottom output fluid channels end up having a much higher concentration of live cells and the center channel contains most of the dead cells.

**Figure 8:**
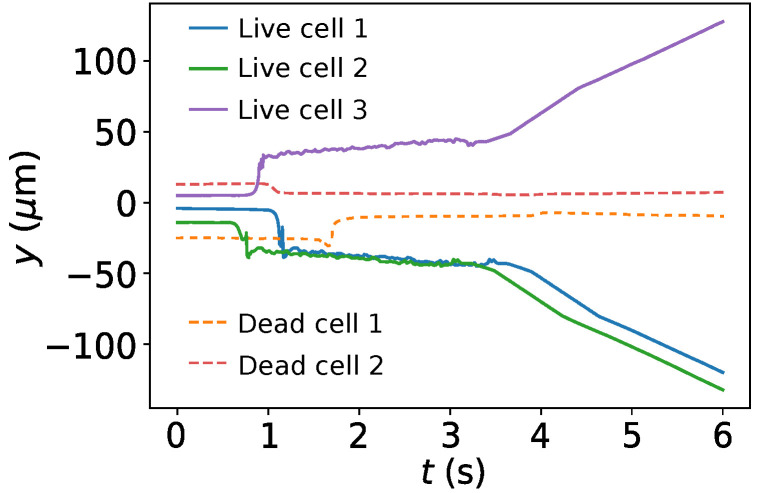
*y* trajectory data of three live cells and two dead cells.

The sorting mechanics can be analyzed further by plotting the trajectory of a few cells, as shown in Figure 8. During 0<t<0.8 s, the *y* position of the all the cells remain constant as they are not yet near the electrodes to experience the dielectrophoretic force. At this stage, driven by the fluid flow, their motion is limited to the *x* direction. During 0.8 s<t<3.5 s, the cells are within the region of influence of the electrodes. The live cells move toward the high field region (the region between the electrodes) and dead cells move away from there. Since the y=0 line is at the middle of the two high field regions (note Figure 6c), the dead cells accumulate around this line. On the other hand, the live cells accumulate near the top or bottom electrodes. At t>3.5 s, each cell enters one of the three output microfluidic channels. The fluid flows in the top and bottom channels change directions creating a shift in cell trajectory. This can be noted in Figure 8 as the live cells follow a trajectory parallel to the top or bottom channels. The dead cells follow a constant *y* path through the center channel. This is consistent with the experimental results reported by Doh et al. in their paper [15]. Thus, the simulation accurately demonstrates the cell sorting or separation mechanics.

## 7. Conclusions

A numerical model that can simulate behavior of particles in LOC devices was developed. Using the model, we successfully simulated the operations of an optical trap and a dielectrophoretic cell sorter. The numerical solver is generally applicable and can simulate most LOC devices once the external force terms are defined. It can be used for the development and optimization of new and existing LOC devices. The solver was coded using open-source Python libraries and can be run on freely accessible compilers. We also made our code for the solver publicly available [26]. Due to the general nature, the developed solver can be of interest to researchers working on biomicrofluidics and LOC devices.

## Data Availability

The data that support the findings of this study are available from the corresponding author upon reasonable request.

## Supplementary Materials

The following are available online at https://www.mdpi.com/article/10.3390/mi12101265/s1, The Supplementary Material contains a detailed description of Clausius–Mossotti factor and how the dielectric function of cells were modeled. Multimedia files showing animation of the simulations are also included in the supplementary materials.
