# Supplementary material for: Modeling Brownian Microparticle Trajectories in Lab-on-a-Chip Devices with Time Varying Dielectrophoretic or Optical Forces
Source: Micromachines (Basel). 2021 Oct 18;12(10):1265. doi: 10.3390/mi12101265 (PMC8539052; doi:10.3390/mi12101265)
Supplement: Supplementary file 1 [file micromachines-12-01265-s001.zip › Supplementary/supplementary.pdf]

Supplementary Information: A python platform for simulating Brownian dynamics in lab-on-a-chip devices

[1,*], Mohammad Asif Zaman[1,*], Mo Wu[1], Punnag Padhy[1], Michael A. Jensen[2], Lambertus Hesselink[1], and Ronald W. Davis[2,3]

[1]Department of Electrical Engineering, Stanford University, Stanford, CA 94305, USA
[2]Stanford Genome Technology Center, Department of Biochemistry, Stanford University, Palo Alto, CA 94304, USA
[2]Department of Genetics, Stanford University, Palo Alto, CA 94304, USA
[*]zaman@stanford.edu

Clausius-Mossotti factor and Dielectric Function of Cells

The dielectrophoretic force equation (under dipole approximation) contains the frequency dependent Clausius-Mossotti factor, $\tilde{C}_M(\omega)$. Here $\omega = 2\pi f$ is the angular frequency of the AC electric field (f is the frequency in Hz). The ˜ symbol over a variable indicates that it is a complex number. We note that the sign of $\Re\left[\tilde{C}_M(\omega)\right]$ determines the direction of the force. Positive $\tilde{C}_M(\omega)$ gives positive DEP (i.e. particles attracted to high electric field regions) and negative $\tilde{C}_M(\omega)$ results in negative DEP (i.e. particles repelled from high electric field regions). $\tilde{C}_M(\omega)$ is a measure of the effective polarizability of the particle in the medium. It is defined as[1]:

$$\tilde{C}_M(\omega) = \frac{\tilde{\varepsilon}_c(\omega) - \tilde{\varepsilon}_m(\omega)}{\tilde{\varepsilon}_c(\omega) + 2\tilde{\varepsilon}_m(\omega)}. \tag{S1}$$

Here $\tilde{\varepsilon}_{m,c}(\omega)$ is the complex dielectric functions of the materials. The subscripts m and c represent the suspension medium and the cell (yeast), respectively. The complex dielectric function of the medium is modeled as:

$$\tilde{\varepsilon}_m(\omega) = \varepsilon_m - i\frac{\sigma_m}{\omega\varepsilon_0} \tag{S2}$$

where ε_m is the relative permittivity, and σ_m is the conductivity of the medium. We use $\varepsilon_m = 80$ and $\sigma_m = 0.8 \times 10^{-3}\,\text{S/m}$ in our calculations[2]. Eq. S2 holds for homogeneous isotropic particles/objects.

Cells have more complex structures than homogeneous particles. They are composed of different distinct materials (cytoplasm surrounded by a thin cell membrane). Thus, a more complex approach is necessary to model $\tilde{\varepsilon}_c$. A shell-model is often used to model the effective dielectric function of cells[3–5]. According to this model, $\tilde{\varepsilon}_c$ is given by[1]:

$$\tilde{\varepsilon}_\text{c} = \tilde{\varepsilon}_\text{mem} \frac{\left(\frac{r_2}{r_1}\right)^3 + 2\frac{\tilde{\varepsilon}_\text{cyt} - \tilde{\varepsilon}_\text{mem}}{\tilde{\varepsilon}_\text{cyt} + 2\tilde{\varepsilon}_\text{mem}}}{\left(\frac{r_2}{r_1}\right)^3 - \frac{\tilde{\varepsilon}_\text{cyt} - \tilde{\varepsilon}_\text{mem}}{\tilde{\varepsilon}_\text{cyt} + 2\tilde{\varepsilon}_\text{mem}}}, \tag{S3}$$

where $\tilde{\varepsilon}_\text{cyt}$ and $\tilde{\varepsilon}_\text{mem}$ have the same form as Eq. S2 and represent the complex dielectric function of the cytoplasm and membrane, respectively, and r_1 and r_2 are the inner and outer cell radii, respectively. The material properties of the cytoplasm and cell membrane is needed to evaluate the dielectric function of a cell. The conductivity, relative permittivity and the geometric parameters of live and dead yeast cells have been reported in the literature[2,6]. By plugging in these values, it is possible to calculate the Clausius-Mossotti factor of live and dead yeast cells using Eq. S1, S2, and S3. The results are shown in Fig. S1. It can be noted that the frequency response of a live cell is significantly different than that of a dead cell. It is possible to find frequency regimes when their responses have opposite signs. For the cell sorter/separator LOC considered in this paper, the frequency of interest is $f = 5\,\text{MHz}$. We can note that at that frequency, $\Re\left[\tilde{C}_M(\omega)\right] > 0$ for live cells (viable) and $\Re\left[\tilde{C}_M(\omega)\right] < 0$ for dead cells (nonviable). The exact numbers are: $\Re\left[\tilde{C}_{M,\,\text{viable}}\right] = 0.945$ and $\Re\left[\tilde{C}_{M,\,\text{nonviable}}\right] = -0.25$. Thus, for an AC electric field with frequency 5 MHz, the live cells will experience positive DEP forces where as the dead cells will experience negative DEP forces. This is consistent with results found in literature[5,7]. Due to the opposing DEP forces, it is possible to separate the two types of cell.

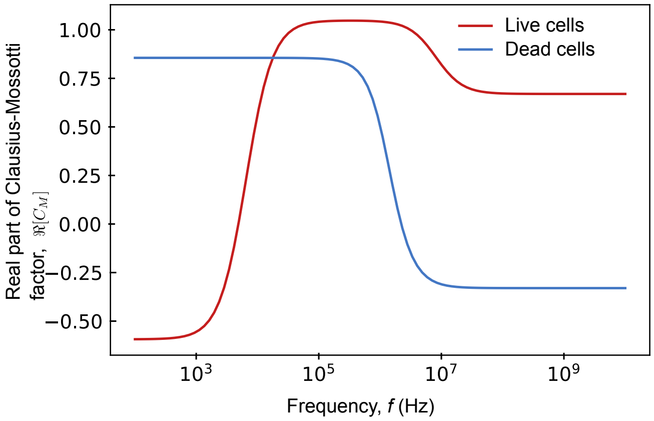

Figure S1. Real part of the Clausius-Mossotti factor for live and dead yeast cells.

References

1. Pethig, R. Review article—dielectrophoresis: Status of the theory, technology, and applications. *Biomicrofluidics* **4**, 022811 (2010). DOI 10.1063/1.3456626.
2. Talary, M. S., Burt, J. P. H., Tame, J. A. & Pethig, R. Electromanipulation and separation of cells using travelling electric fields. *J. Phys. D: Appl. Phys.* **29**, 2198–2203 (1996). DOI 10.1088/0022-3727/29/8/021.
3. Gagnon, Z. R. Cellular dielectrophoresis: Applications to the characterization, manipulation, separation and patterning of cells. *ELECTROPHORESIS* **32**, 2466–2487 (2011). DOI 10.1002/elps.201100060.
4. Adams, T. N. G., Turner, P. A., Janorkar, A. V., Zhao, F. & Minerick, A. R. Characterizing the dielectric properties of human mesenchymal stem cells and the effects of charged elastin-like polypeptide copolymer treatment. *Biomicrofluidics* **8**, 054109 (2014). DOI 10.1063/1.4895756.
5. Ettehad, H. M. & Wenger, C. Characterization and separation of live and dead yeast cells using CMOS-based DEP microfluidics. *Micromachines* **12**, 270 (2021). DOI 10.3390/mi12030270.
6. Asami, K., Hanai, T. & Koizumi, N. Dielectric properties of yeast cells. *The J. Membr. Biol.* **28**, 169–180 (1976). DOI 10.1007/bf01869695.
7. Doh, I. & Cho, Y.-H. A continuous cell separation chip using hydrodynamic dielectrophoresis (DEP) process. *Sensors Actuators A: Phys.* **121**, 59–65 (2005). DOI 10.1016/j.sna.2005.01.030.
